# The profile of extreme tension wave front in aluminum

**DOI:** 10.1007/s10853-023-08244-6

**Published:** 2023-02-07

**Authors:** Seokbin Lim, James Kennedy, Angel Chavira, Matthew Hirsch, Tie Wei, Donghyeon Ryu

**Affiliations:** New Mexico Tech, Socorro, NM 87801 USA

## Abstract

In this study, the extreme tension wave front profile (pull speed up to 1.6 km/s) in pure aluminum (density 2.7 $\mathrm{g}/{\mathrm{cm}}^{3}$) is analyzed using the LAMMPS molecular dynamics (MD) code and based on the tension conservation equations of mass, momentum, and energy. The simulation results agree favorably with the theoretical calculation. The profile of the extreme tension wave front is observed from the MD code simulation, and a typical shockless ramp wave front formation is identified during forced extreme tension loading. Further analysis was accomplished based on the formation of the ramp wave front, illustrating the behavior of the isentrope of aluminum under extreme tension loading.

## Introduction

The conservation equations based Rankine–Hugoniot jump equation has led to an understanding of the behavior of materials under extreme compression. This set of equations represents the fundamental relation among the shock properties (under one-dimensional uniaxial strain condition), including the pressure, density, particle velocity, and internal energy profiles behind a steady shock, and this has been used as a standard approach for extreme dynamic compressive conditions, Eqns. (1), (2), (3) [1, 2].

1 $$U{\rho }_{0}=\left(U-{u}_{p}\right)\rho$$

2 $$P-{P}_{0}=\rho U{u}_{p}$$

3 $$E-{E}_{0}=\frac{1}{2}\left(P+{P}_{0}\right)\left({v}_{0}-v\right)$$

The Rankine–Hugoniot jump equations require more inputs, however, to close the equations. This is because it is an underdetermined equation with five unknown parameters in three equations. An additional empirical Hugoniot plane (or $U-{u}_{p}$) has to be applied to complete the set of equations, and other shock-parameter correlations (i.e.,$P-v, P-{u}_{p}$) have been built from this. The Hugoniot state is the locus of end states under steady shock compression in extreme pressure (i.e. up to 120 GPa in Al), and various modifications and analyses have been made, leading to the construction of equations of states (EoS) of materials under extreme compressive conditions [2–4].

Due to the complex nature of material behavior under extreme dynamic compression, the use of highly sophisticated empirical test methods and theoretical analysis have been the main approaches to investigations in this area. The use (or invention) of various data-acquisition systems (such as VISAR or PDV) and the development of high-velocity impact test configurations has expanded experimental possibilities over the last several decades [5–9]. High-velocity impact testing has been shown to be a viable method for understanding material behavior under extreme compressive conditions.

Because the Hugoniot is a collection of final states not properly representing the thermodynamic path [1, 2], a new empirical test-based analysis has been developed. This includes the quasi-isentrope of material properties based on isentropic compression experiments (ICE) using various techniques. This approach aims to create a shockless gradual ramp wave rather than a sudden shock jump in the sample by the use of a graded density impactor or an electromagnetically driven system [6, 7, 10, 11]. This technique, which should produce a relatively small viscous flow effect, aims to achieve a thermodynamic isentrope of materials during compressive incidents. This technique will not produce an exact isentrope since the impact-based test configuration will create irreversible friction heat on the molecular level, so it is referred to as quasi-isentropic. Given the absence of a steady state, the following formulae have been built based on the local Lagrange (or in situ) fluid-flow assumption along the ramp wave front [7, 10–12].

4 $$d{\sigma }_{x}={\rho }_{0}\cdot {C}_{L}({u}_{p})\cdot d{u}_{p}$$

5 $$dv=\frac{du}{{\rho }_{0}\cdot {C}_{L}({u}_{p})}$$

This approach differs from the conventional shock-based analysis, in that it is based on the shockless gradual ramp wave loading of the target and seeks to evaluate local conditions during wave propagation. This allows study of the thermodynamic path (or quasi-isentrope) under extreme compression [7, 10–12].

In opposite to the extreme compressive nature of materials, there have been numerous researches regarding extreme tensile condition. This is mostly aiming to understand tensile fracture (i.e. spallation). Starting from the experimental observation of spalling behavior [13–15], there has been a gradual evolution of the theory. This includes understanding of the growth and nucleation of micro-cracks [16], and determination of tensile fracture criteria in terms of time-dependent stress conditions during tensile loading [17, 18]. There has been considerable research on spall strength, based on various assumptions. One notable approach to the study of the spall strength was proposed by Novikov in 1966, as follows [19]:

$$\sigma =\frac{1}{2}{\rho }_{0}{C}_{0}\Delta {u}_{fs}$$

This is a linear approximation of spall strength based on the so-called velocity pullback deduced from experimental data. This approach has been further developed and modified to improve the accuracy, and the determination of tensile strength using this simple equation is still considered to be a viable method [19].

Most previous approaches to spalling, however, are based on empirical observation or data fit. Furthermore, data collection is limited to the sample surface while the real fracture occurs inside the sample, and this can lead to misinterpretation of the nature of tensile fracture. The difficulty is further complicated by the nature of spalling, which is driven by shock reflection at the free surface. The initial shock contaminates the area where the fracture occurs or alters the sample property prior to the arrival of the spall signal. These issues may produce questionable results as to the tensile behavior of the sample, and prohibit accurate prediction of behavior under extreme tension loading. In some cases, indeed, extrapolated data from compressive incidents has been used [19]. These issues are mainly driven by the lack of scientific study about the extreme dynamic tension wave propagation. For example, the conventional shock Hugoniot data only cover the dynamic compression and there is no information about the negative (or tension) side. For this reason, the fundamental mechanisms (or material behaviors) under extreme dynamic tension loading is urgently required for further progress.

Molecular dynamics (MD) simulations of single crystal metals, with fine time scales and realistic physics, are well established for very high strain rate hydrodynamic studies. These have been developed over the last 30 years, and include shock response of materials [20–22]. Through these MD techniques, Jarmakani et al. resolved disagreements on materials undergoing shockwaves [23], and similar atomistic studies have been carried out by Argawal et al. [24], that realistically replicate the two-wave (elastic–plastic) structure above the Hugoniot elastic limit (HEL) with the embedded atom method (EAM). In general, there has been considerable research on the use of MD code for extreme compressive conditions, and the results have converged well with the experimental and theoretical data. Almost all of the research and publication on tensile behavior-related MD simulation, on the other hand, have been about spall behavior or deformation or crack formation at the molecular level during tensile loading, without consideration of the nature of the tensile wave.

The lack of information about the extreme dynamic tension wave has limited much scientific investigation prohibiting in-depth understanding of the general material behavior under the extreme environment. Since many extreme dynamic events occur under the tension regime, i.e. high-velocity expansion, fracture, crack, spall, etc. (or compression and tension occur simultaneously), a proper scientific investigation about the extreme tension wave propagation is necessary.

As a part of this effort, we aim to understand the behavior of materials under extreme dynamic tension loading, based on the conservation equations. Numerical data from LAMMPS (Large-scale Atomic/Molecular Massively Parallel Simulator) code simulations of pure aluminum (density 2.7 $\mathrm{g}/{\mathrm{cm}}^{3}$) will be presented in order to evaluate the theoretical approach. An aluminum sample was selected to realize the extreme tension wave in this paper due to its popularity in various space and military applications where the extreme tensile environment is quite common (i.e. spacecraft fuel tank rupture, fragmentation of explosives casing, etc.), expecting that the similar behavior can be observed in other material samples.

## Extreme dynamic tension model (conservation equations)

**Figure 1:**
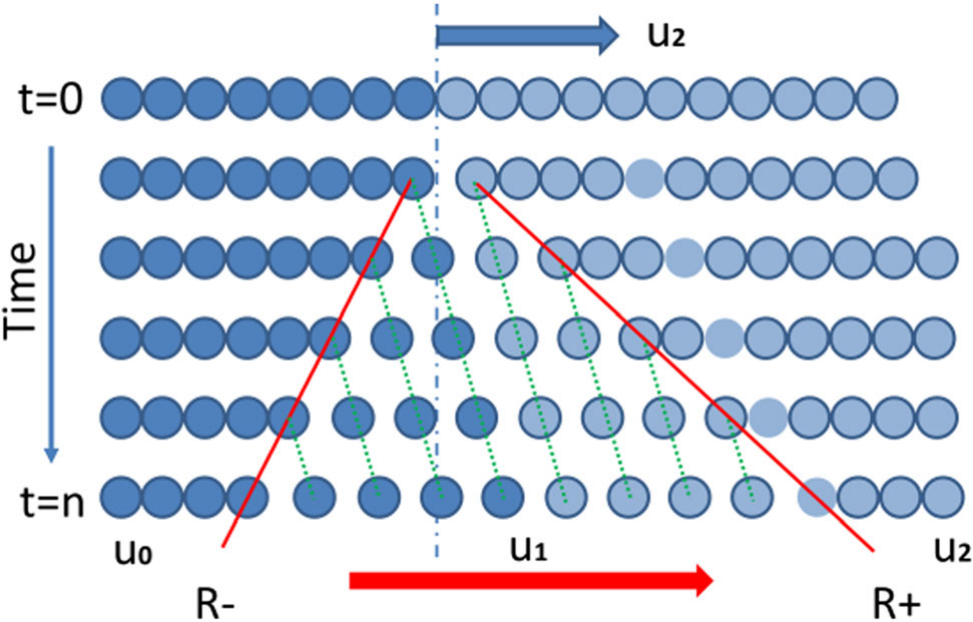
A string of beads under tension for each time step

The behavior of material under extreme tension loading can be illustrated by analogy with a string of beads (with the intermolecular attraction). Consider a string of beads under a sudden pull speed of ${u}_{2}$ on the entire half right side of the string (Fig. 1). As soon as the string is pulled, the center beads are pulled apart, creating tension waves in ${R}^{-}$ and ${R}^{+}$ toward the undisturbed material in opposite directions. This makes the beads between ${R}^{-}$ and ${R}^{+}$ areas move in the positive direction with ${u}_{1}$. The beads under ${u}_{1}$ are under dynamic tension, and this is the area of our interest.

**Figure 2:**
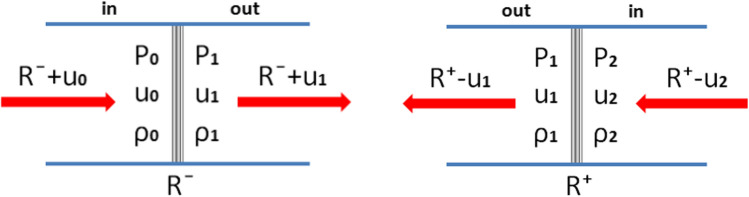
Schematic of tension waves (Left:${R}^{-}$, Right: ${R}^{+}$)

When we consider the tension waves ${R}^{-}$ and ${R}^{+}$ separately in Eulerian coordinates (a standing tension wave, and the material flows through the wave), the following two schematic diagrams can be built (Fig. 2) [25].

From Fig. 2, the following conservation equations for each region of ${R}^{-}$ and ${R}^{+}$ can be built, where the subscripts o, 1 and 2 represent the initial, tension, and pulled area conditions individually.

Mass conservation for ${R}^{-}$:

$${\rho }_{0}\left({R}^{-}+{u}_{0}\right)={\rho }_{1}\left({R}^{-}+{u}_{1}\right)$$

We assume ${\mathrm{u}}_{0}=0$, and the equation is simplified as follows:

6 $$\frac{{\rho_{0} }}{{\rho_{1} }} = \frac{{R^{ - } + u_{1} }}{{R^{ - } }}\;{\text{or}}\;\rho_{1} = \frac{{\rho_{0} R^{ - } }}{{R^{ - } + u_{1} }}$$

Momentum conservation for $R^{ - }$:

$${P}_{1}-{P}_{0}={\rho }_{1}\left({R}^{-}+{u}_{1}\right){ u}_{1}-{\rho }_{0}\left({R}^{-}+{u}_{0}\right) {u}_{0}$$

or

$$P_{1} = \rho_{1} \left( {R^{ - } + u_{1} } \right)u_{1} ,$$

where $\left( {P_{0} = u_{0} = 0} \right)$, and by combining it with the mass conservation,

7 $$\therefore {P}_{1}={\rho }_{0}{R}^{-}{u}_{1}$$

Mass conservation for ${R}^{+}$:

$${\rho }_{1}\left({R}^{+}-{u}_{1}\right)={\rho }_{2}\left({R}^{+}-{u}_{2}\right)$$

or

$${\rho }_{1}={\rho }_{2}\frac{{R}^{+}-{u}_{2}}{{R}^{+}-{u}_{1}}$$

Momentum conservation for $R^{ + }$:

$$P_{1} = \rho_{1} \left( {R^{ + } - u_{1} } \right)u_{1} - \rho_{2} \left( {R^{ + } - u_{2} } \right)u_{2} ,$$

where $P_{2} = 0$, and from the mass conservation,

8 $$\therefore {P}_{1}={\rho }_{2}\left({R}^{+}-{u}_{2}\right)\left({u}_{1}-{u}_{2}\right)$$

By combining the two sets of equations using ${R}^{-}={-R}^{+}+{u}_{2}$ (or ${R}^{+}={-R}^{-}+{u}_{2}$), the following relation can be constructed:

9 $$\therefore u_{1} = \frac{1}{2}u_{2} ,\;{\text{where}}\;\rho_{0} = \rho_{2}$$

From these equations, we see that when a material is physically pulled at a speed of ${u}_{2}$, the particle velocity in the region of tension (${u}_{1}$) should be around one half of ${u}_{2}$. This is because the tension particle momentum is divided into two different directions, ${R}^{+}$ and ${R}^{-}$, and this is quite similar to the relation between the particle velocity and the free surface velocity where $u\approx \frac{1}{2}{u}_{fs}$ in a typical compressive shock propagation. Note that typical compressive shock conservation equations are built on the shock propagation in the target side without consideration of the impactor side. If the same approach (both target and impactor side) is applied to the compressive shock conservation, one can deduce the same result.

In terms of energy conservation, the following equation for ${R}^{-}$ side can be attained.

10 $$E-{E}_{0}=\frac{1}{2}\left({P}_{1}+{P}_{0}\right)\left({v}_{1}-{v}_{0}\right)$$

In summary, the conservation equations for dynamic tension were built based on an assumption that the tension wave front is a single-step (or sudden jump) wave structure, and that the equations are identical to the conventional compressive conservation equations (or Rankine–Hugoniot equations) of Eqs. (1), (2) and (3), except for the use of ${R}^{+}$ and ${R}^{-}$. This set of theoretical equations should apply to any case. The following section will address the same pull condition in a MD simulation, and the result will be compared to the conservation equations above.

## MD (LAMMPS) simulation of extreme tension

Rarefaction differs from tension in the sense that rarefaction occurs in natural relaxation while tension occurs under an externally forced pull. There have been numerous studies in the characteristics of rarefaction. The objective of the present work is to understand the material behavior under forced pull (or tension) without initial shock propagation, which may disrupt the sample properties. In order to understand typical material behavior and tension wave propagation under forced pull, the molecular dynamics code LAMMPS is used. The objective of this numerical study is to understand the detail of the extreme tension wave profile and to ensure the accuracy of the conservation equations against the result from the numerical analysis. A MD code was selected as a numerical tool for this study in order to prevent the data inaccuracy driven by the limited input sources. This is because a typical MD code is rather simple and straight forward not requiring other input sources (i.e. strength model, EoS, failure model, erosion, etc.) which are quite common for hydro-code modeling. By the use of a MD code, one can approach closer to the natural behavior of materials without depending on the quality of other input sources.

LAMMPS is a classical MD code for simulating molecular or atomic interaction. The use of an MD code makes sense in this application because inter-molecular properties are the only factors that determine the (as yet unknown) extreme tension properties during the event, and so will help to illuminate the nature of tension wave.

In the present work, the EAM potential was used for single-crystal aluminum, as developed by Mishin et al. [26]. A simple rectangular bar with approximately 90 × 30 × 30 nm^3^ with 3.2 M atoms of aluminum of density of 2.7 $\mathrm{g}/{\mathrm{cm}}^{3}$ was constructed using the LAMMPS platform [27]. The axial length of the sample bar was determined to be long enough not to create wave-interference at the location of interest. The lattice constant is 4.05 with FCC (face centered cubic) and mono-crystalline configuration. We use the mono-crystalline configuration to create an idealized pulling event and avoid considering the directional anisotropies of the sample, and to prevent the crystal direction-dependent Hugoniot relation, which is outside the scope of this work.

**Figure 3:**
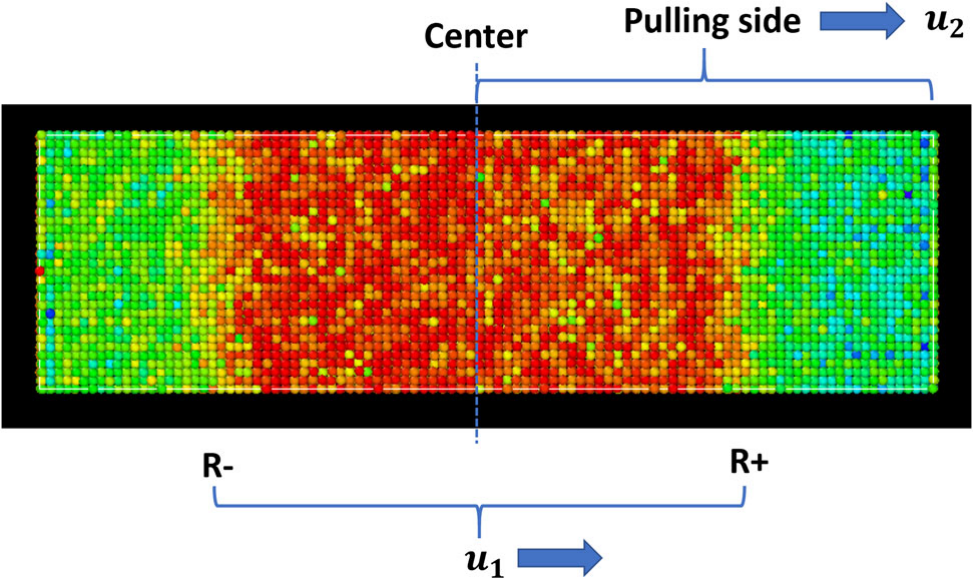
LAMMPS model setup

In order to realize the 1D and uniaxial strain condition (or to eliminate edge effects or necking and release wave), periodic boundaries were used in the lateral direction (top and bottom in Fig. 3). The boundary is free (shrink-wrapped) in the direction normal to the wave. All dynamic simulations were computed with the model temperature at 75 K (with NVE ensemble). This temperature was chosen in order to prevent severe thermal vibration of atoms from interfering with observations of the structure under strain [23] while the initial temperature is not too close to 0 K. The model was equilibrated for 0.2 ns to ensure that residual internal stresses were kept to a minimum. Only one half of the right side of the model was initially pulled in an axial direction (or [100] direction) with no other fixed points, in such a way as to create the ideal pulling condition under the theoretical approach addressed in the section above (Fig. 3).

To investigate sample behavior and tension wave propagation, the sample was pulled at velocities in the range ${u}_{2}$= 500–1600 m/s and the data were collected every 100 fs (femtosecond). Typically, the simulation ran around 7000 fs, or enough time to analyze the apparent rupture or failure behavior without any fixed atoms. Due to the fluctuation of the collected data for each atom, an average value of around 10 atoms in a specific location of interest was chosen.

It was observed that the sample ruptures at around 1500 m/s in the given potential and configuration. Because this study is not aiming to understand the fracture behavior and a typical MD code is not accurate in prediction of fracture occurrences, the data points near the fracture were removed from the analysis. This will eliminate the axial directional release wave (originated from the fracture) from the study, and to focus only on the pure tension front in an ideal configuration. Note that this analysis targets the dynamic tension behavior before formation of the crack; after formation of the crack, the release wave will negatively affect the integrity of the data.

**Figure 4:**
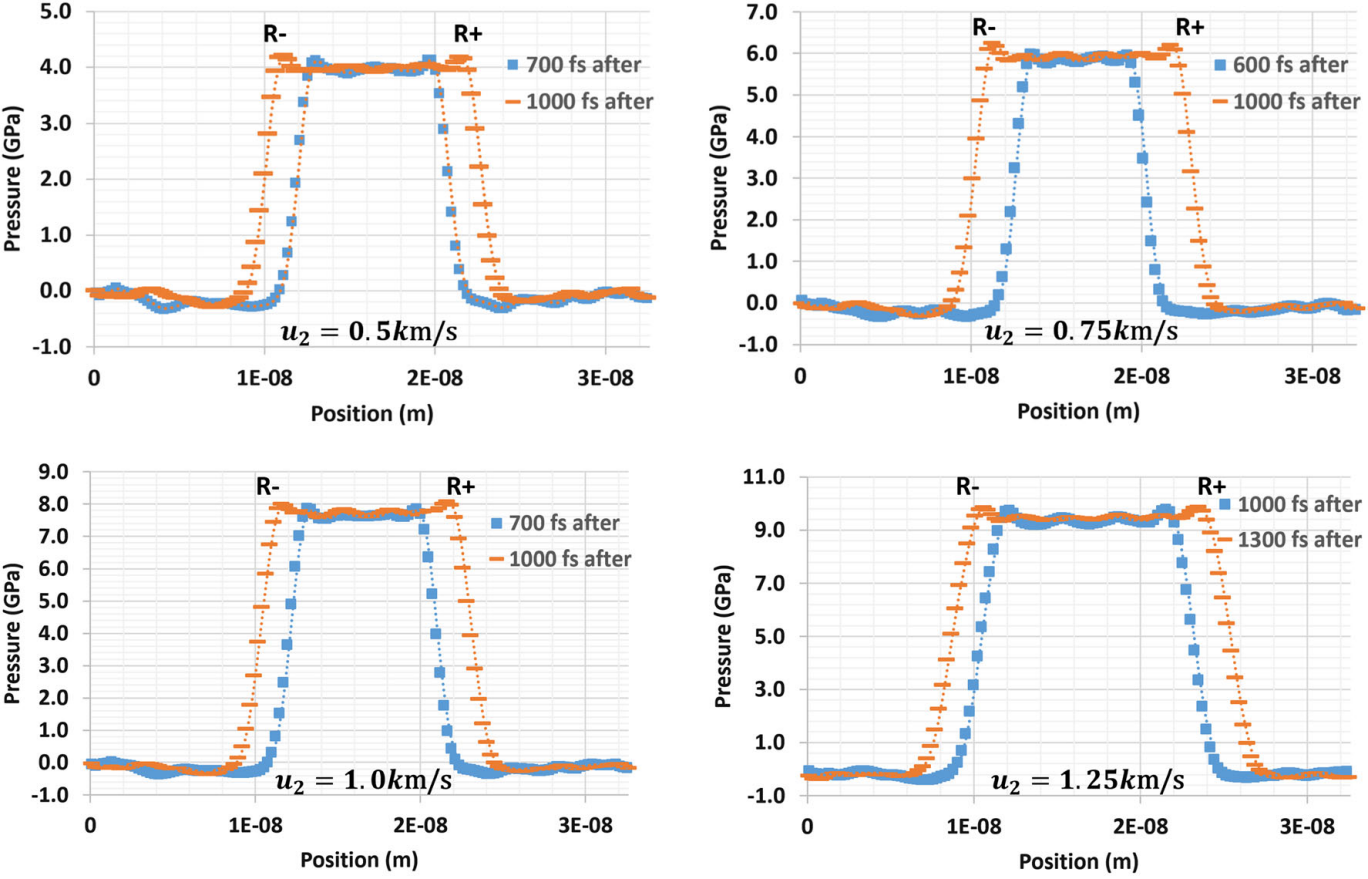
Tension wave pressure profile for various pull speeds (${u}_{2}$) in the range 0.5–1.25 km/s. Each graph shows two different time steps in the entire sample bar length after the pull begins, to suggest the evolution of wave profile and pressure. 0 m. is the left end and 3e-08 m. is the right end of the sample bar

The following graphs show the wave profile during the tension event in terms of pressure for various ${u}_{2}$ (Fig. 4).

It is obvious that the tension pressure increases as the pull speed ${u}_{2}$ increases. There is a smooth increase (or a ramp profile) of pressure at the beginning of the pull (discussed later), and a high tension plateau (or peak) pressure is maintained between ${R}^{-}$ and ${R}^{+}$ while the pull is continued until fracture occurs (the moment of fracture is intentionally removed from the graph in order to focus in the given topic).

From the pressure profiles above, it is clear that the tension wave does not experience a distinct sudden jump. The formation of the tension wave is different from the typical compressive shock, because the tension wave is related to the particle pulling interaction, whereas the transfer of the tension wave always depends on the in situ speed of sound between particles (or atomic attraction), where the atomic repulsive force is not applicable (no physical impact or repulsive force between particles). That is, when the shock pressure is large enough (or when the particle velocity increases), the particle velocity (as opposed to the local speed of sound) is dominant.

As a first step in studying the steady-state regions in the plots above, we assume that the tension wave front is a single-step structure wave. The following data are collected from the plateau (or the peak) area in various pull speeds and compared with the theoretical calculations (Table 1).

**Table 1:** Tension data: LAMMPS and theory (conservation eqns.)

Pull speed (km/s)	Particle velocity (km/s)	LAMMPS results^1^			Theoretical calculation		
		Density (g/cc)	Tension wave velocity (km/s)	Pressure (GPa)	Density (g/cc)	Pressure (GPa)	Specific volume (cc/g)
${u}_{2}$	${u}_{1}$	${\rho }_{1}$	${R}^{-}$	${P}_{1}$	${\rho }_{1}$	${P}_{1}$	${v}_{1}$
0	0	2.700^3^	0	0	2.700	0	0.370
− 0.50	− 0.250	2.590	6.200	− 4.200	2.595	− 4.185	0.385
− 0.60	− 0.300	2.570	6.250	− 5.000	2.576	− 5.063	0.388
− 0.70	− 0.350	2.550	6.190	− 5.750	2.556	− 5.850	0.391
− 0.75	− 0.375	2.538	6.000	− 6.100	2.541	− 6.075	0.394
− 0.80	− 0.400	2.530	6.120	− 6.500	2.534	− 6.610	0.395
− 0.90	− 0.450	2.510	5.900	− 7.200	2.509	− 7.169	0.399
− 1.00	− 0.500	2.488	5.800	− 8.000	2.486	− 7.830	0.402
− 1.10	− 0.550	2.470	5.600	− 8.500	2.459	− 8.316	0.407
− 1.20	− 0.600	2.445	5.375	− 9.100	2.429	− 8.708	0.412
− 1.35	− 0.675	2.411	5.200	− 9.900	2.390	− 9.477	0.418
− 1.50^2^	− 0.750	2.373	4.800	− 10.600	2.335	− 9.720	0.428
− 1.60^2^	− 0.800	2.350	4.700	− 11.100	2.307	− 10.152	0.433

^1^Data were collected over 0.5–2.5 ps (picosecond) and averaged

^2^These data points have been removed from the analysis due to the fracture of the sample

^3^Initial density of aluminum, ${\rho }_{0}$

The theoretical calculation is carried out using the tension conservation equations above (Eqs. 6, 7) based on the measured speed of the tension wave (${R}^{-}$) at the peak area. The speed of the tension wave in the peak area was measured based on the location of the ${R}^{-}$ front at two different time steps in the MD code simulation. Once this value is collected, the rest of values of the theoretical calculation can be obtained. Note that the speed of tension wave needs to be measured experimentally (a typical compressive shock speed is also an empirically measured value), and the construction of governing equation for the speed of tension wave is out of scope in this paper.

**Figure 5:**
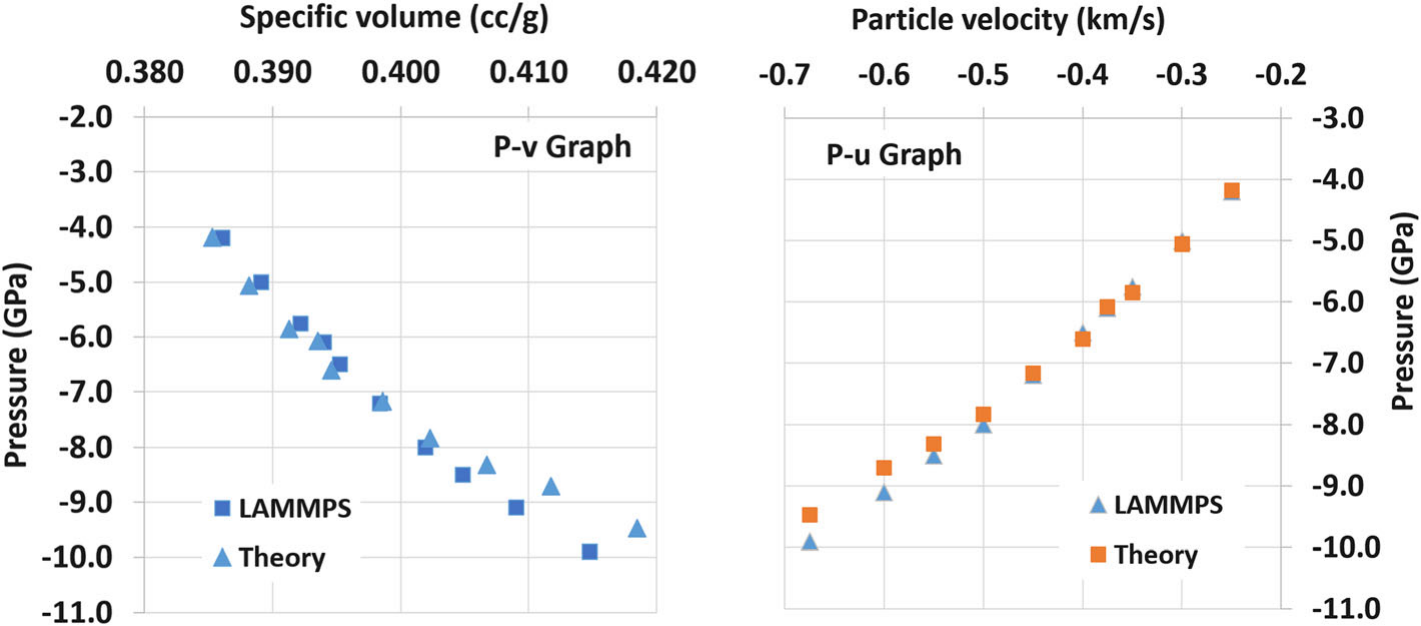
Tension wave properties (refer to Table 1)

The theoretical density of the sample during the tension was calculated using Eq. (6), and the data from Table 1 are plotted in Fig. 5.

$${\rho }_{1}=\frac{{{\rho }_{0}R}^{-}}{{R}^{-}+{u}_{1}}$$

**Figure 6:**
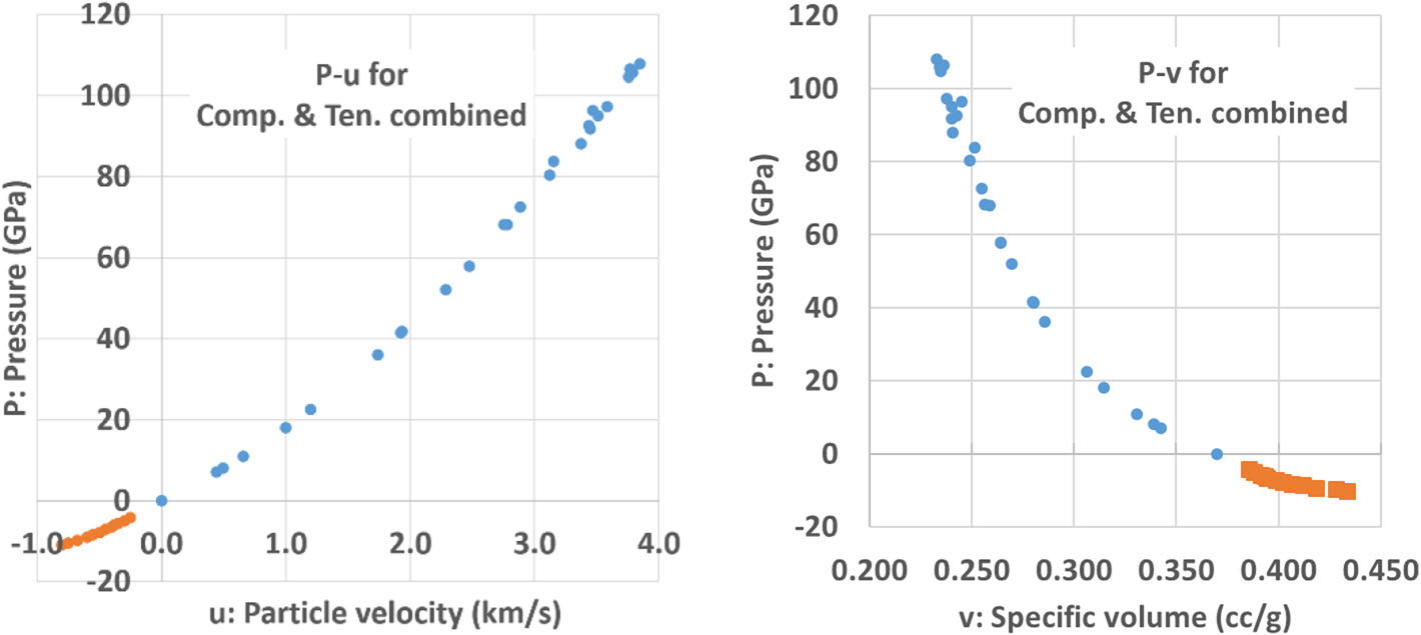
Combination of compression Hugoniot (P–v) and peak tension values [28].* Note* 1: the compression Hugoniot is based on Al6061 [28], and the tension (points with negative pressure with orange colored dots) is based on aluminum with density 2.7 $\mathrm{g}/{\mathrm{cm}}^{3}$.* Note* 2: Al6061 was used for the comparison due to the similarity of the density with the given Al sample in the simulation

From Fig. 5, it is possible to see the general tendency of the tension wave in the given range. Both graphs show some discrepancy (less than ~ 0.5 GPa) between the LAMMPS results and the conservation equation-based calculation in the high tension pressure area (below − 8 GPa), and there is good agreement in low tension pressure area in general (− 4 GPa ~ − 8 GPa). The discrepancy in the high pressure area (between − 8 and − 10 GPa) is believed to originate from the selection of groups of atoms in the peak pressure area where the tension wave velocity is measured. The current tension curves are similar to the collection of multiple peak tension states (or tension Hugoniot). It is not clear at this point whether this series of data should be considered as a simple collection of the final states (no thermodynamic path) or rather as a thermodynamic path during the tension wave propagation that is similar to the isentropic path in the ramp wave propagation. However, based on the fact that the tension conservation equations are identical to the compression cases (or Rankine–Hugoniot), the two curves are combined, and show a smooth continuity (Fig. 6) [28].

The above analysis is related to the profile and nature of the tension wave; a more detailed study was conducted to understand the ramp wave profile of the tension wave front.

## Lagrange tension wave (tension isentrope)

**Figure 7:**
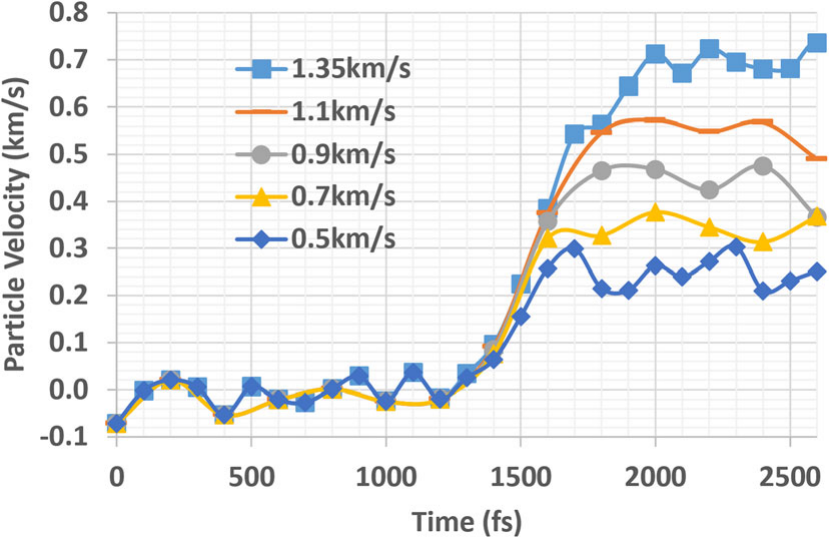
Variation of particle velocity (${u}_{1}$) depending on pull speed (${u}_{2}$)

The vibrational nature of the atoms in LAMMPS tends to obscure the unique nature of tension wave propagation. In order to overcome this difficulty, a group of atoms was averaged in a specific location (or bin) of interest during tension wave propagation. As a first step, the particle-velocity profile during the tension event was collected for five different pull speeds ${u}_{2}$, in the range 0.5–1.35 km/s (Fig. 7).

As we addressed above in the theoretical analysis, the particle velocity is around one half of the pull speed of the sample. There is considerable fluctuation in the data over time, but the relation between the particle velocity and the pull velocity agrees favorably with the equation, ${u}_{1}=\frac{1}{2}{u}_{2}$.

**Figure 8:**
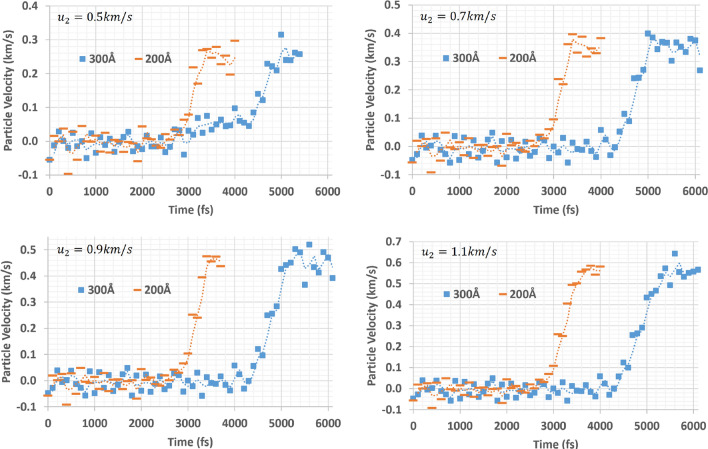
Tension wave front profiles at two different locations from the center

The profile of the tension wave front presents some unique characteristics. Due to the nature of the MD code, which considers miniscule time periods (fs: femtoseconds) and atomic sizes (Å: Angstrom or 1 Å = 0.1 nm), the initial slope angle in the wave front may be considered as a sudden jump and we need to see the detailed trend of the wave front. In order to understand the nature of the tension wave front profile, the wave front profile of ${R}^{-}$ at different distances (200 Å and 300 Å) from the center are collected and compared (Fig. 8). Two wave front profiles were collected for each pull speed.

The figures above show the evolution of the tension wave front as it travels. Overall, there is no indication of a sudden particle velocity jump or two-step structure (an initial elastic precursor followed by a plastic tension wave). The elastic precursor portion (speed around ~ 6.3 km/s) can be seen in the lower particle velocity range by analysis of the speed of the wave [11, 29] (Fig. 10), but there is no abrupt separation from the following plastic wave portion. Rather, the elastic precursor and the following plastic wave are smoothly coupled, demonstrating a typical ramp wave structure. Furthermore, the maximum particle velocity portion (or peak plateau area) is maintained over the given range of time before fracture occurs without any noticeable fluctuation.

**Figure 9:**
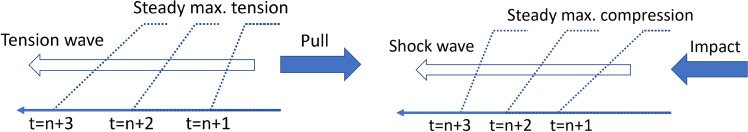
Evolution of wave (difference between the extreme tension and shock)

The tension wave front clearly shows a unique profile where the slope of the ramp wave front decreases as the wave travels. In other words, the evolution of the tension wave front is opposite to the shock front formation, in the sense that the unique sudden jump of a shock front originates from an initial ramp wave propagation [6, 7, 10, 30] (Fig. 9).

Ramp wave formation under tension is mainly driven by the change of the local (or in situ) wave speed (or Lagrange wave speed) in different particle velocity, and as the particle velocity increases, the Lagrange wave speed decreases.

**Figure 10:**
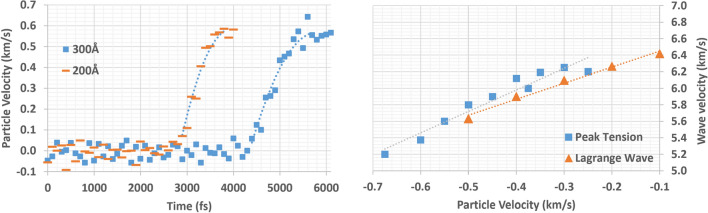
Lagrange wave velocity

In order to understand the evolution of local Lagrange wave speed, the detailed wave front profile of ${u}_{2}=1.1 \mathrm{km}/\mathrm{s}$ is studied (Fig. 10). Based on the wave profile of ${u}_{2}=1.1 \mathrm{km}/\mathrm{s}$, a smooth trend line along the data points is created to collect the Lagrange wave speed for each particle velocity (Table 2).

**Table 2:** Lagrange wave profile

Particle velocity	Location at 200Å^2^	Location at 300 Å	dt^3^	Lag. Vel. (*C*L)	Lag vel. (*C*L)	Tension pressure
km/s	fs^1^	fs	fs	100 Å/fs	km/s	Gpa
0	2823.44	4348.85	1525.41	0.06556	6.55561	0.000
− 0.1^4^	2924.02	4482.81	1558.79	0.06415	6.41523	1.732
− 0.2	3035.68	4632.00	1596.32	0.06264	6.26441	3.383
− 0.3	3163.15	4803.20	1640.05	0.06097	6.09738	4.939
− 0.4	3315.99	5010.56	1694.57	0.05901	5.90120	6.373
− 0.5	3520.94	5296.68	1775.74	0.05631	5.63146	7.602

^1^Femtosecond

^2^200 Å away from the point of pulling

^3^Time difference when the wave reaches 200 Å and 300 Å from the pulling point

^4^Negative values represent the particle moves left side

**Figure 11:**
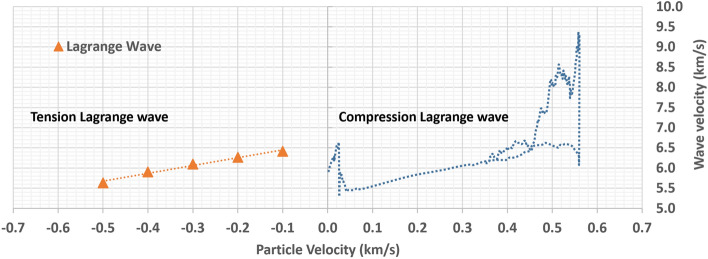
Lagrange wave speeds for tension and compression [11, 29]

The Lagrange wave velocity profile of Al6061 has been discussed in several publications on the subject of compression [11, 29]. Although it is not possible to compare the result directly to this tension data, we were able to see favorable agreement between the two. A simple comparison of the slope angle of the Lagrange wave velocities for compression and tension reveals that an identical slope angle and similar initial elastic precursor speed in early stage of loading delivers meaningful results (Fig. 11). However, the current tension Lagrange wave profile collected from the MD code does not show the distinct elastic (in lower particle velocity region) or quasi-elastic (high particle velocity region) features that are quite typical in compression. This may be as a result of the difference between computational simulation and experiment.

**Figure 12:**
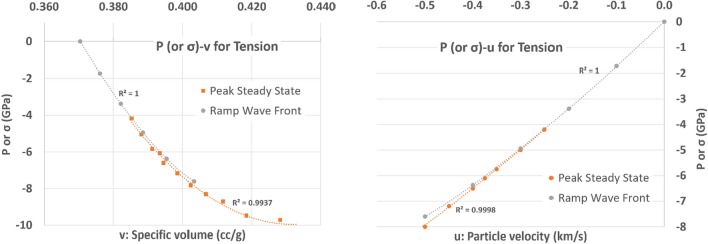
Tension P–v and P–u planes in the peak steady state and ramp wave front

Based on the data collected from LAMMPS in the ramp wave front, P–v and P–u curves are developed and are compared to the data in the peak plateau area (or steady state) as follows (Fig. 12).

We separate the data for the ramp wave front and the peak steady state because the ramp wave front is closely related to the isentrope of the material under extreme loading. Another words, this ramp wave profile will provide the information about the thermodynamic property (no Hugoniot path) of the materials under extreme condition. In addition, since this condition does not require a high velocity impact where the viscous flow is inevitable, it may deliver more accurate thermodynamic properties. The peak steady state, however, would indicate the Hugoniot state, where the irreversible and adiabatic condition exists. From the figures above, we can conclude that there is a clear difference between the peak steady state and the ramp wave front. The difference is negligible when the loading pressure is low, but when the pressure increases, the difference approaches close to 0.4–0.5 GPa when the loading pressure is close to 8 GPa. This aspect may contribute the discrepancy of ~ 0.5 GPa in Fig. 5, but this is not clearly identified yet. In the given range of data and the given simulation configuration, we conclude that there is clear difference between the two curves indicating viscous flow (irreversible condition with increase in entropy) of the sample material during sudden tension loading. More in-depth analysis of the thermodynamic properties during sudden tension loading is required.

## Discussion

A MD code simulation result varies by the given potential and various atomic configuration of the sample. Because the MD code analysis in the context above is based on a selected potential and the atomic configuration, more in-depth study with the use of other potentials will be necessary to ensure the quality of the result. This is because the nature of MD code is limited by the selected potential, and the potential has to make sense within the context that they were applied. For example, if the potentials were not made for studying thermal responses then their applicability is limited because the vibrational nature (or kinetics) of atoms is closely related with temperature.

In a MD simulation, temperature is calculated by the following equation, $\mathrm{KE}=n\cdot \frac{aDOF}{2}\cdot k\cdot T$, where KE is the total kinetic energy, n is the number of atoms, aDOF is the degrees of freedom per atom, *k* is Boltzmann's constant, and *T* is temperature. Solving for *T* we see that only the velocity of the atoms affects the temperature via KE.

**Figure 13:**
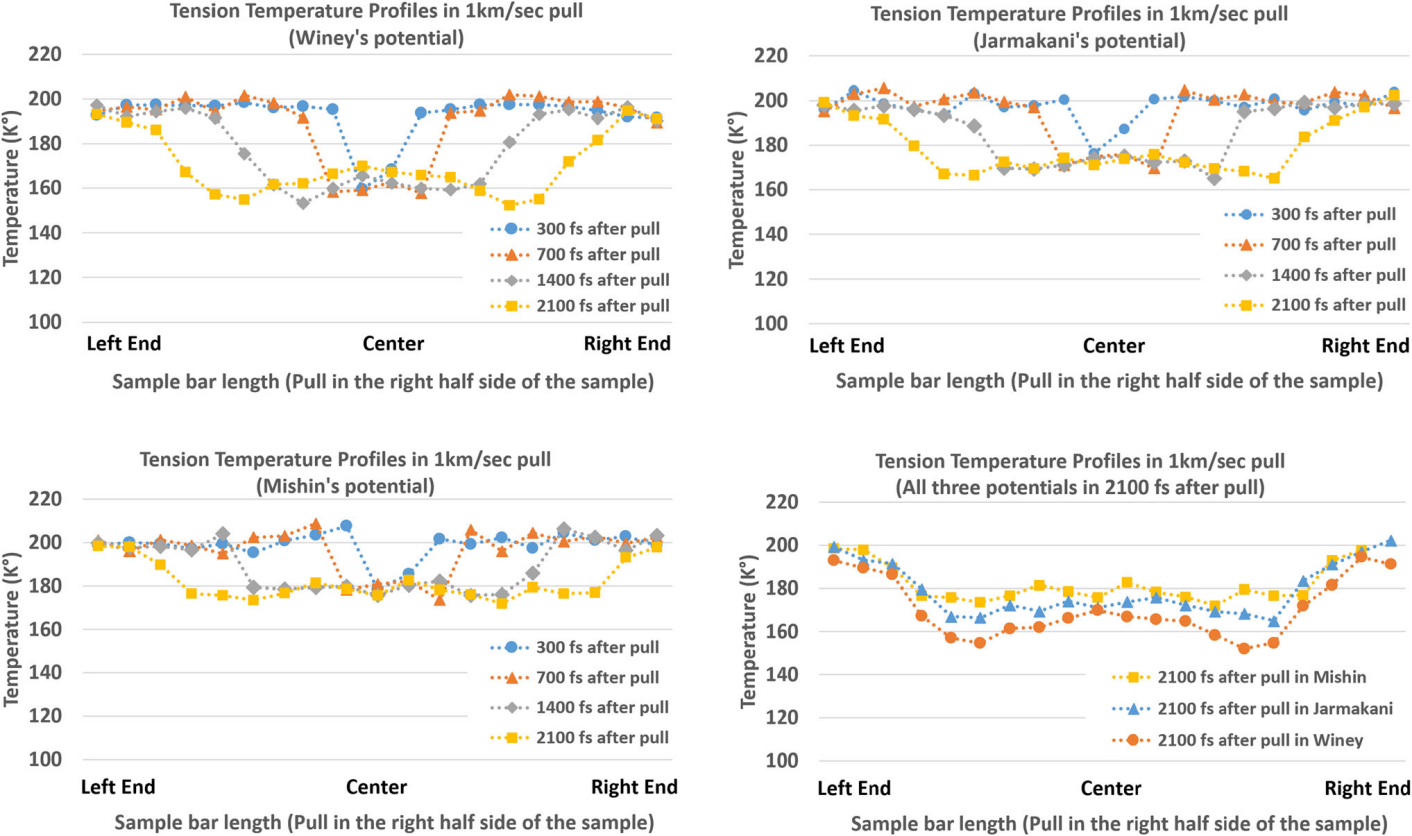
Temperature profiles depending on the different potentials

As a part of this analysis, the temperature profile during the tension (initial temperature is set to 200 K in all three potentials) were compared between three different potentials of Winey, Jarmakani and Mishin [23, 26, 31] (Fig. 13).

All three different potentials commonly show temperature decrease during the tension. In addition, all three potentials show a ramp wave pattern at the front of the tension wave, and the slope angle of the tension wave front (ramp wave) gets lower as it propagates. The change in the slope angle of the ramp wave is not compared in the current study, but all three potential shows a slightly different slope angle change.

**Figure 14:**
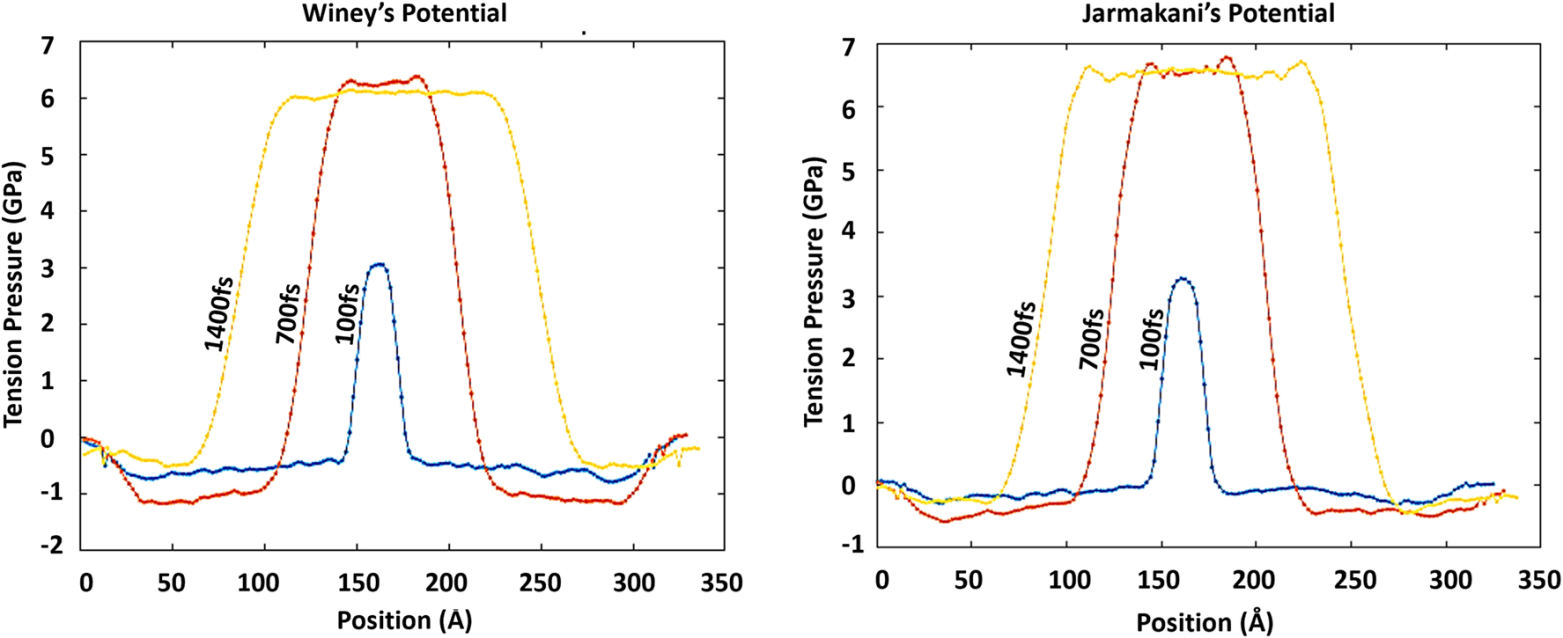
Tension pressure (1 km/s pull speed) in Winey’s and Jarmakani’s potentials (Note that the tension pressure is in the positive side of the graph)

In terms of temperature, all three potentials show a different temperature profile. Winey’s potential shows the lowest temperature profile range (minimum 154 K° at 2100 fs) and Mishin’s potential shows the highest (minimum 171 K° at 2100 fs). All three potentials show the minimum temperature right at the tension wave front (or peak point of the ramp). After this point, each potential shows a different pattern of temperature change. Mishin’s potential predicts the temperature behind the wave front maintains a similar temperature without much fluctuation. However, Winey’s potential shows temperature increases right behind the tension wave front. It wasn’t clear at this point why each potential shows such different temperature profile behind the wave front, but this is closely related with the molecular kinetic (per each potential) right behind the tension wave front. In more details, Winey’s potential also shows the lowest tension wave speed (4.538 km/s) in all potentials indicating the lowest tension pressure prediction (approx. pressure range of 5.97–6.15 GPa, fluctuating pressure) (Fig. 14 and Table 3).

**Table 3:** Tension pressure comparison

Potential	Initial Aluminum density (g/cm^2^)	Wave travel distance in 700fs^1^ (Å)	Tension wave speed (km/s)	Particle velocity (km/s)	Tension pressure in the code (GPa)	Theoretical tension pressure (GPa) (Eq. 7)
Winey’s	2.7	31.77	4.538	0.5	5.95–6.15^2^	6.126
Jarmakani’s	2.7	34.78	4.968	0.5	6.45–6.75	6.707

^1^Femtosecond

^2^All tension pressure is described as positive values

^3^Mishin’s potential is not listed in this table since this is the selected potential in the analysis above

Note that Winey’s potential shows 31.77 Å of wave propagation distance in 700 fs (between 700 and 1400 fs after pull starts). Based on Eq. (7), one can calculate the tension pressure of 6.126 GPa (${\rho }_{0}=2.7 \mathrm{g}/{\mathrm{cm}}^{3}$) which is similar to the collected tension pressure from the simulation. This simple comparison indicates that the proposed theoretical analysis can still be applicable in all three different potentials even though their predicted range of values are all different, indicating the reasonable repeatability and applicability of the proposed analytical model in various potentials and results.

In general, the MD simulation results agree favorably with the proposed conservation equations-based theoretical calculations (Eqs. 6 ~ 9), and the general characteristics of tension wave in aluminum under the extreme tension loading have been identified. One of the unique findings from this analysis is the formation of a ramp wave front during tension wave propagation. The gradual slope of the ramp wave front is distinctively different from the compressive shock front, in which the speed of the wave at highest pressure overruns the elastic precursor, ${C}_{\mathrm{L}}$, in the lower pressure area. The tension wave, however, exhibits behavior opposite to that of the compressive shock. When the tension pressure increases, the speed of the wave at the peak point decreases while ${C}_{\mathrm{L}}$ maintains the constant value. This creates a typical (or gradual) ramp wave led by the elastic precursor (6.3–6.5 km/s in Al). This behavior is also evident in Fig. 6, the P–v curve, where the Rayleigh line, $({P}_{2}-{P}_{1})/({v}_{2}-{v}_{1})$, on the tension side is always negative, and the slope of the Rayleigh line gradually decreases as the tension pressure increases. The Rayleigh line is related to the local wave speed, which can explain why the tension wave forms a ramp wave front.

One of the limitations of the present analysis is the lack of information regarding the attenuation of the tension wave (or the end of tension wave). The MD code simulation reveals steady-state tension loading before the fracture occurs. Since the focus of this paper is not the prediction of the fracture of the sample, the MD code simulation was run until fracture occurred, and further analysis was not performed. We believe that the fracture of the sample is affected by the duration (or impulse) of the steady-state tension pressure. Once the fracture occurs after the passage of enough tension impulse (followed by the propagation and coalescence of micro-cracks, and so on), the tension-release wave from the fracture surface will propagate from behind the tension wave front, leading to dissipation of the tension wave. Because the sample was in tension before the arrival of the tension-release wave, the tension-release wave may be related to the compressive nature where the particles get close to (or collide) each other in high velocity. At the same time, the slope angle of the ramp wave front decreases as it propagates, creating another attenuation of the steady-state peak tension loading from the front. Clearly, further analysis is required.

## Conclusions

MD simulation associated with the conservation equations provides a way to understand the typical forced tension wave propagation in aluminum material with density 2.7 $\mathrm{g}/{\mathrm{cm}}^{3}$, focusing on the pure tension wave front prior to tensile fracture. This analysis shows good agreement between the MD code results and the theoretical prediction.

One of the unique findings of this analysis is the identification of the formation of the ramp wave front during sudden tension loading, and the decrease in the slope of the ramp wave front during propagation. It is well known that this kind of evolution occurs in the typical rarefaction wave as a part of the shock attenuation process [1, 2, 4], but here we demonstrate that the forced tension wave front shows a similar phenomenon. The reduction in slope angle usually occurs as a part of the attenuation process during compressive shock propagation, but the current result shows that the reduction in the slope angle in the tension wave actually occurs at the front. The extreme tension wave and the compressive shock wave behave in opposite fashion.

Based on the formation of the ramp wave front during extreme tension loading, more in-depth analysis was conducted to identify the difference between the peak steady state and the ramp wave front. The analysis indicates the nature of the tension wave propagation, where thermodynamic properties can vary depending on the state of the wave propagation.

## List of symbols

$$U$$: Shock velocity
$R$: Tension wave velocity
$$\rho$$: Density
$${u}_{\mathrm{p}}(or u)$$: Particle velocity
${u}_{\mathrm{fs}}$: Free surface velocity
$E$: Specific internal energy
$$P$$: Pressure
σ: Stress
${C}_{0}$: Bulk speed of sound
$$v$$: Specific volume
${C}_{\mathrm{L}}$: Lagrange wave speed
